# Minocycline corrects early, pre-plaque neuroinflammation and inhibits BACE-1 in a transgenic model of Alzheimer's disease-like amyloid pathology

**DOI:** 10.1186/1742-2094-9-62

**Published:** 2012-04-02

**Authors:** Maria Teresa Ferretti, Simon Allard, Vanessa Partridge, Adriana Ducatenzeiler, A Claudio Cuello

**Affiliations:** 1 Department of Pharmacology and Therapeutics, McGill University, 3655 Promenade Sir-William-Osler, Room 1210, Montreal, QC H3G 1Y6, Canada; 2 Department of Anatomy and Cell Biology, McGill University, Montreal, QC H3A 2B2, Canada; 3 Department of Neurology and Neurosurgery, McGill University, Montreal, QC H3A 2B4, Canada

**Keywords:** Alzheimer, Aβ-oligomers, BACE, iNOS, Microglia, Minocycline, NFkB

## Abstract

**Background:**

A growing body of evidence indicates that inflammation is one of the earliest neuropathological events in Alzheimer's disease. Accordingly, we have recently shown the occurrence of an early, pro-inflammatory reaction in the hippocampus of young, three-month-old transgenic McGill-Thy1-APP mice in the absence of amyloid plaques but associated with intracellular accumulation of amyloid beta petide oligomers. The role of such a pro-inflammatory process in the progression of the pathology remained to be elucidated.

**Methods and results:**

To clarify this we administered minocycline, a tetracyclic derivative with anti-inflammatory and neuroprotective properties, to young, pre-plaque McGill-Thy1-APP mice for one month. The treatment ended at the age of three months, when the mice were still devoid of plaques. Minocycline treatment corrected the up-regulation of inducible nitric oxide synthase and cyclooxygenase-2 observed in young transgenic placebo mice. Furthermore, the down-regulation of inflammatory markers correlated with a reduction in amyloid precursor protein levels and amyloid precursor protein-related products. Beta-site amyloid precursor protein cleaving enzyme 1 activity and levels were found to be up-regulated in transgenic placebo mice, while minocycline treatment restored these levels to normality. The anti-inflammatory and beta-secretase 1 effects could be partly explained by the inhibition of the nuclear factor kappa B pathway.

**Conclusions:**

Our study suggests that the pharmacological modulation of neuroinflammation might represent a promising approach for preventing or delaying the development of Alzheimer's disease neuropathology at its initial, pre-clinical stages. The results open new vistas to the interplay between inflammation and amyloid pathology.

## Background

Alzheimer's disease (AD) is a devastating neurodegenerative condition affecting more than 35 million people worldwide [1]. Neuropathological examination of the brains of AD patients reveals intraneuronal neurofibrillary tangles (composed of paired filaments of abnormally phosphorylated tau protein [2]), and massive accumulation of extracellular amyloid plaques composed of aggregated amyloid beta peptide (Aβ) [3].

The initiating event for Aβ production is the cleavage of the amyloid precursor protein (APP) by the β site APP cleaving enzyme 1 (BACE-1), a neuronal specific aspartyl protease [4]. This event generates a soluble N-terminus exodomain (soluble APPβ) liberated into the lumen and a β-C-terminus fragment (β-CTF) bound to the membrane. Gamma secretase cleavage of the membrane-anchored β-CTF releases Aβ peptides of different lengths, including Aβ38, Aβ40 and Aβ42 [5]. Aβ42 readily aggregates into neurotoxic oligomers and eventually forms mature fibrils and plaques [6]. Amyloid plaques in humans and animal models are invariably accompanied by activated astrocytes and microglia with elevated levels of pro-inflammatory products [7].

While Aβ accumulation and aggregation are considered central events in the AD neuropathology, the mechanisms that underlie these processes remain to be elucidated. In particular, the role of neuroinflammation in the progression of the disease is a matter of intense debate. There is increasing awareness that the inflammatory response in neurodegeneration is a highly dynamic process [8]. In AD, most studies have focused on the late, plaque-associated glial activation; this phenomenon has been the object of extensive investigations and it has been well-characterized in the human brain, in several animal models and in *in vitro *settings [7]. While fibrillar Aβ-stimulated microglia are capable of secreting toxic factors *in vitro *[9], peri-plaque microglia appear to elicit mostly beneficial effects *in vivo*, limiting plaque growth by phagocytosing Aβ and releasing neurotrophic factors [10,11]. In agreement with such observations, prospective clinical trials with anti-inflammatory drugs in patients with AD have shown no effect, or even a worsening of the pathology [12-14]. On the other hand, epidemiological data demonstrated that life-long users of nonsteroidal anti-inflammatory drugs (NSAIDs) develop AD with reduced frequency. This association suggests the existence of a latent pre-clinical inflammatory process which would facilitate the disease progression (for a review, see [15]).

Besides the epidemiological studies, a growing body of evidence in the literature supports the concept that inflammation is an early event in the progression of AD. Microglial activation could be detected in patients with mild cognitive impairment (MCI), which represents the prodromal stage of AD [16-19]. Furthermore, gliosis and up-regulation of IL-1β have been reported in fetal and neonate patients with Down syndrome [20]. Since individuals with Down syndrome invariably develop plaque pathology by mid-age, prenatal and neonatal samples can be considered as pre-plaque conditions.

Taken together, the available evidence strongly indicates that microglial activation occurs early in the progression of the disease. It is very likely that the glial response at the first stages of the neurodegenerative process differs significantly from the well-established peri-plaque inflammation, and could accelerate the onset of the disease. Unfortunately, direct investigation of microglial activation and its role in pre-clinical stages of AD is complicated by the fact that it is impossible to predict the conversion of individuals with no cognitive impairment into individuals with MCI or AD. Therefore, very little is known about the status of microglial activation and its role in the earliest, pre-clinical stages of AD.

In this regard, transgenic (Tg) animal models, which faithfully recapitulate the main hallmarks of the AD-like amyloid pathology, offer the opportunity to investigate events associated with the progression of the disease. It is becoming increasingly clear that Tg mice with extensive plaque deposition but without neurofibrillary tangles and neuronal loss represent a relatively early stage of the pathology compared to a human brain and are most likely models of early AD and MCI [21]. Accordingly, pre-plaque APP-Tg mice can be valuable tools to elucidate even earlier events preceding plaque deposition.

Using this approach, we have recently described the occurrence of an early, pre-plaque inflammatory response in our newly generated McGill-Thy1-APP mouse Tg model of AD-like amyloid pathology that would mimic the pre-morbid AD. In this Tg model, plaque deposition begins around four to five months of age [22]; however, up-regulation of inflammatory markers and activation and mobilization of microglia can be detected as early as three months of age [23]. Indications of pre-plaque inflammation have also been reported for other models such as APP(V717) [24], R1.40 [25] and 3xTg [26]. However, the role of such early neuroinflammation in the progression of the amyloid pathology had not yet been determined.

To define the role of the early, pro-inflammatory events observed at pre-plaque stages of the AD-like amyloid pathology, we tested the therapeutic effect of minocycline, a tetracyclic derivative with anti-inflammatory properties, in young, pre-plaque McGill-Thy1-APP Tg mice. The treatment lasted one month and ended when the mice were three months old, thus prior to the appearance of the first plaques. This strategy allowed us specifically to investigate the role of inflammation in early, pre-plaque stages of the amyloid pathology, which should correspond to the earliest, pre-clinical stages in the human. We gathered biochemical and morphological evidence indicating that early, pre-plaque neuroinflammation can be blocked by minocycline treatment. The reduction of inflammatory markers was accompanied by reduced activity of BACE-1 and correction of the nuclear factor-kappa-light chain enhancer of activated B cells (NFkB) pathway.

## Materials and methods

### Animals and treatment

For these studies we used our in-house APP Tg mouse model of AD-like amyloid pathology, coded McGill-Thy1-APP [22]. The mice carry the human APP transgene with the Swedish and the Indiana mutations under the control of the murine Thy1.2 promoter. All the animals were two months old when they started the treatment, and were sacrificed at three months of age. Young, pre-plaque two-month-old Tg mice received minocycline by intraperitoneal injection for one month (Tg Mino, n = 7); control groups included vehicle-treated Tg mice (Tg Placebo, n = 7), vehicle-treated non Tg, age-matched littermates (Non Tg Placebo, n = 8) and non Tg, age-matched littermates treated with minocycline (Non Tg Mino, n = 8). All the animals received intraperitoneal injections of 200 uL of solution, alternating the injection side, every day for one month. Minocycline hydrochloride was purchased from Sigma-Aldrich Canada (M9511; Oakville, ON, Canada) and a fresh solution of 5 mg/mL (10 mM) was prepared in filtered PBS and stored at -80°C for one week. Since the pH of minocycline hydrochloride is acidic (pH 4), we corrected it to neutrality by adding sodium hydroxide (as described by [27]). Minocycline-treated animals received a dose of 50 mg/Kg/day (200 μL of 5 mg/mL solution equating to 1 mg, which, for an average mouse weighing 20 g, corresponds to 50 mg/Kg). This dose is similar to previous studies [28] and is expected to result in micromolar concentrations of the drug in the brain [29]. Placebo animals received an equal volume of filtered PBS, pH 7.4.

The animals were housed in groups of up to four in individually ventilated cages under standard conditions (22°C, 12 h light-dark cycle) receiving food and water *ad libitum*. All procedures were approved by the Animal Care Committee of McGill University and followed the guidelines of the Canadian Council on Animal Care. After three weeks of the treatment, one Non Tg Mino mouse had to be euthanized for a swollen intestine and two Tg Mino mice died (no statistical difference was found in the mortality across genotypes, *P *= 0.59, chi-squared test). No mice died in the placebo groups. At the end of the treatment the animals (Non Tg Placebo: n = 8, six females and two males; Tg Placebo: n = 7, five females and two males; Non Tg Mino: n = 7, six females and one male; Tg Mino: n = 5, two females and three males) were sacrificed by transcardial perfusion and the brains processed for biochemical and immunohistochemical analysis.

### Perfusion and tissue preparation technique

Tg and Non Tg littermate mice were deeply anesthetized with equithesin (pentobarbital-based, 2.5 mL/Kg, intraperitoneal injection) and perfused through the heart with ice-cold saline solution (pH 7.4) for 1 minute. The brains were then quickly removed and divided into right and left hemisphere on ice. The cortex, hippocampus and cerebellum were dissected from the left hemisphere, snap-frozen in dry ice and stored at -80°C for biochemical analysis. Cortical samples were used for the determination of inflammatory marker levels and APP-related products, while hippocampi were used for BACE-1 activity assay and BACE, NFkB and inhibitor of kb (IkB) quantification. The right hemisphere was fixed in 4% paraformaldehyde in 0.1 M phosphate buffer (pH 7.4) for 24 hours at 4°C. The tissue was then cut into 40-μm thick sections with a freezing sledge microtome (SM 2000R, Leica, Wetzlar, Germany) and free-floating sections were collected in PBS and processed for immunohistochemistry.

### Western blotting

#### *Inflammatory markers*

Cortical samples from the left hemisphere were homogenized in 250 μL of lysis buffer (50 mmol/L Tris-HCl, 150 mmol/L sodium chloride, 1% Nonidet P-40, 0.1% sodium dodecyl sulfate, 0.1% deoxycholic acid, 2 μg/mL of aprotinin, 2 μg/mL of leupeptin, 100 μg/mL phenylmethanesulfonyl fluoride, pH 7.4). The samples were centrifuged at 13,000 rpm for 45 minutes at 4°C. Following total protein content quantification (Dc-protein assay, Bio-Rad, Hercules, CA, USA), 100 μg of protein were separated using 10% SDS-PAGE and semi-dry transferred to nitrocellulose membranes for subsequent western blotting. Membranes were blocked with 5% non-fat milk in Tris-buffered saline (TBS) containing 0.1% Tween 20 (TBS-T) and then incubated with the primary antibody overnight at 4°C. Primary antibodies used were rabbit polyclonal anti-inducible nitric oxide synthase (iNOS) and IL-1β (both 1:500; Santa Cruz Biotechnology Inc., CA, USA); cyclooxygenase-2 (COX-2; 1:2000; Cayman Chemicals, Ann Arbor, MI, USA); and mouse monoclonal anti βIII-tubulin (1:40,000; Promega, Madison, WI, USA). Horseradish peroxidase (HRP)-conjugated anti-rabbit and anti-mouse secondary antibodies were purchased from Jackson (Jackson Immunoresearch Laboratories, West Grove, PA, USA). The HRP signal was revealed with a chemiluminescence assay (ECL, GE Healthcare, Amersham, UK) on films. Signal intensity was quantified by densitometry (MCID4 image analysis system, Imaging Research Inc., St. Catherine's, ON, Canada). The levels for each marker were normalized with respect to βIII-tubulin (neuronal specific) immunoreactivity. All experiments were performed in triplicate.

#### *Tris-tricine western blotting for amyloid precursor protein and amyloid precursor protein-related products*

Total proteins (100 to 250 μg) from the cortical homogenates (prepared as indicated above) were run in pre-cast commercially available 10 to 20% Tris-tricine gels (Criterion, Bio-Rad Laboratories). The proteins were semi-dry transferred on nitrocellulose (for 6E10 detection) or polyvinylidene fluoride (for pab27576) for 2 hours at 12 V. The membranes were boiled for 5 minutes in PBS, then blocked for 2 hours with milk 10% and incubated with the specific antibody over night at 4°C. Antibodies used were monoclonal mouse antibodies 6E10 (1:1000; directed against the residues 1 to 16 of human Aβ; from Signet, provided by Covance, Princeton, NJ, USA) and neuron specific βIII tubulin (1:40,000; Promega); and rabbit polyclonal pab27576 (1:250; directed against the C-terminus of APP; a generous gift from Dr Multhaup, Freie University, Berlin). The quantification was performed as described above; for CTF, the duplet was quantified. For the calibration curve, a recombinant CTF (C100 protein purified recombinantly from Escherichia coli) was used (generous gift from Dr Multhaup). The C100 had a C-terminal hexa-His tag and an N-terminal start-methionine. The calibration curve appeared to be linear (r^2 ^= 0.99) in the range between 7 and 0.43 ng per lane.

#### β-site APP cleaving enzyme 1, nuclear factor kappaB and inhibitor of kb

The hippocampi were briefly sonicated in 100 μL of Cell Extraction Buffer (provided with the BACE activity kit, see below), incubated on ice for 15 minutes and centrifuged at 10,000 rpm for 5 minutes at 4°C. The protein content was quantified as described above and 100 μg of total protein was separated using 10% SDS-PAGE and transferred to polyvinylidene fluoride membrane for subsequent western blotting. Membranes were blocked with 5% non-fat milk in TBS-T and then incubated with the primary antibody overnight at 4°C. The primary antibodies used were rabbit polyclonal BACE-1 (PA1-757, 1:250; Thermo Scientific Pierce antibodies, Meridian Road Rockford, IL, USA); NFkB (p65, 1:1000) and IkB (1:2000; both from Santa Cruz Biotechnology Inc.); and actin (1:20,000; Abcam, Cambridge, MA, USA). Band quantification was performed as described above. All experiments were performed in triplicate.

### Immunohistochemistry and quantification of ionized calcium-binding adaptor molecule 1 immunoreactive cells

#### *Bright field immunohistochemistry*

Free-floating immunohistochemical staining was performed as previously described [30,31] using the rabbit polyclonal primary antibody ionized calcium-binding adaptor molecule 1 (Iba-1; 1:10,000; Wako Chemicals USA, Inc., Richmond, VA, USA). For the detection, a biotinylated goat anti-rabbit secondary antibody was applied followed by amplification with the avidin-biotin complex (ABC elite kit, both from Vector Laboratories Inc., Burlingame, California, USA). All stainings were developed with 0.06% 3,3'-diaminobenzidine (Sigma-Aldrich Canada) and 0.01% hydrogen peroxide.

#### *Quantification of ionized calcium-binding adaptor molecule 1-immunoreactive cell soma size and density*

The staining and the quantification of Iba-1 immunoreactive (ir) cells were performed according to published protocols [23]. All the stainings were performed simultaneously. Three sections per animal were chosen in the area corresponding to bregma -2.9 [32], stained with Iba-1 and mounted on gelatinated slides, after which the slides were coded. Digital images were acquired on an Axioplan 2 Imaging microscope (Zeiss, Toronto, ON, Canada), equipped with an AxioCam HRc digital camera (Zeiss), using AxioVision 4 Imaging program (Zeiss). The micrographs were taken with a 63× Zeiss plan-Apochromat oil immersion objective in the CA1 area of the hippocampus (eight micrographs per section, three sections per animal for a total of 24 micrographs per animal,) and, as a control, four micrographs were taken in the lateral posterior thalamic nucleus (total of 12 micrographs per animal). The images were imported into the MCID 5 Image Analysis Software (Imaging Research Inc.) as tagged image file format files and transformed (with the 'target accent' function) to allow optimal detection by the program. Cell bodies of all the cells in the focal plane of each micrograph were manually outlined by a blinded observer before target size, number of target elements, intensity and form factor were measured in an automatic fashion. After quantification, the slides were decoded and the data analyzed. The density data are expressed as the number of Iba1-ir cells per field (38.206 μm^2^). MTF was blind to the nature of the material at all stages of the quantification.

#### *Enzyme-linked immunosorbent assay for human amyloid beta peptide*

Human Aβ40 and Aβ42 levels were quantified from cortical homogenates using a commercially available ELISA kit (Invitrogen, Carlsbad, CA, USA; distributed by Medicorp, Montreal, Canada). Each sample was mixed in an equal volume of guanidine-HCl (to a final concentration of 5 M guanidine) and incubated for 3 hours at room temperature. The resulting samples were further diluted 1:10 in the provided dilution buffer (to a final concentration of 0.5 M guanidine) and tested in duplicate. The amount of Aβ was extrapolated from a calibration curve of synthetic human Aβ40 and Aβ42 using the curve fitting function of Graph-Pad Prism 5 software (La Jolla, CA, USA). The calibration curve was prepared according to the manufacturer's instructions in the presence of 0.5 M guanidine to ensure comparability with experimental samples. The data were normalized on total micrograms of protein content per sample. Controls included: omission of samples (background), chromogenic substrate alone (blank) and Non Tg samples. No signal was detectable in Non Tg samples (data not shown).

#### β-site APP cleaving enzyme 1 activity

We used two commercially available kits for the measurement of BACE-1 enzymatic activity from the biological samples (R&D, Minneapolis, MN, USA and Abcam, Cambridge, MA, USA). The assay was conducted according to manufacturer's instructions. Briefly, hippocampal samples (see above for preparation details) were diluted to a final concentration of 2.5 μg/50 μL. From the resultant samples, 2.5 μg of total protein were loaded into a black 96-well microplate and the fluorogenic substrate was added in the dark. The substrate is conjugated to the EDANS and DABCYL reporter molecules. Cleavage of the peptide by β-secretase separates EDANS and DABCYL, allowing for the release of a fluorescent signal. The reaction was incubated at 37°C for one hour in the dark, and the signal was measured using a Fluostar Optima (BMG Labtech GmbH, Ortenberg, Germany) with 355 nm excitation and 520 nm emission wavelengths. Each sample was run in duplicate; the assay was repeated twice and results from the two experiments were pooled after normalization on Non Tg Placebo values. Negative controls included: omission of the fluorogenic peptide (background fluorescence of the tissue), omission of the sample (blank) and addition of BACE-1 inhibitor to the samples (provided by the kit). We ran 2.5 μg of recombinant human peptide (rhBACE-1: R&D) as a positive control.

### Postnatal day 7 samples for β-site APP cleaving enzyme 1 western blot

Two pups from postnatal day 7 were sacrificed and the brains carefully removed; one brain was immediately homogenized as described above for regular western blotting, while the cortex of the second brain was used to establish a mixed glial culture according to standard protocols with minor modifications [33]. Briefly, the cells were disaggregated using papain (Worthington, Lakewood, NJ, USA) and mild mechanical stress with a polished Pasteur pipette. The cells were then re-suspended in DMEM containing 10% FBS and penicillin-streptomycin, and cultured in a 10-cm petri dish. The medium was changed every three to four days and after two weeks the cells were collected by scraping on ice, then sonicated and processed for western blotting.

### Data analysis

All data were analyzed using the Graph-Pad Prism 5 software (La Jolla). Multiple groups' comparison was done by one-way analysis of variance (ANOVA) followed by Tukey post-hoc test. Kruskal-Wallis was used for non-normally distributed data. The interaction between the genotype and effect of the drug was studied using two-way ANOVA. Correlation studies were done using the Spearman's test for non-normally distributed data. Significance was set at *P *< 0.05. All data are presented as mean ± standard error of the mean.

## Results

### Minocycline corrects neuroinflammation in pre-plaque McGill-Thy1-APP Tg mice

**Figure 1:**
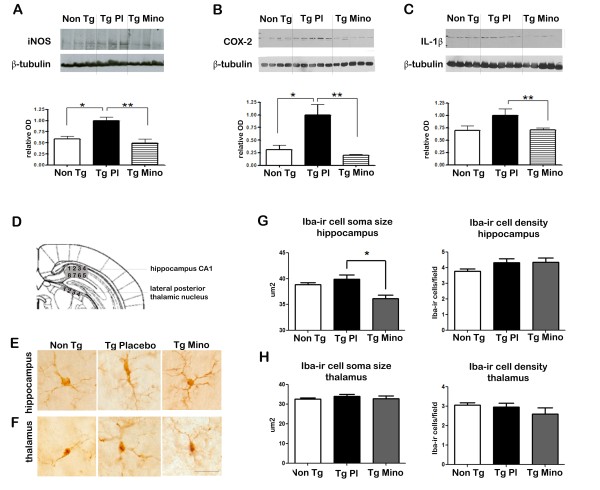
**Minocycline corrects neuroinflammation in young, pre-plaque Tg mice**. **(A-C) **Representative western blots for iNOS, COX-2 and IL-1β (typical markers of microglial and neuronal activation) in control, Non Tg Placebo mice (Non Tg), Tg Placebo mice (Tg Pl) and Tg mice treated with minocycline (Tg Mino). Note the significant up-regulation of iNOS and COX-2 in Tg Pl compared to Non Tg. Minocycline restored iNOS and COX-2 to levels similar to those of Non Tg, and significantly reduced the levels of IL-1β (* *P *< 0.05, **P < 0.01, one-way ANOVA with Tukey post-hoc test). See main text for the values of Non Tg treated with minocycline. **(D) **Schematic illustrating the sampling of images in the CA1 area of the hippocampus and from the lateral posterior thalamic nucleus as utilized for the morphological study represented in E-G. **(E) **Representative micrographs illustrating the ir of Iba-1 in microglial cells in the hippocampus of the Non Tg, Tg Pl and Tg Mino mice. Note the altered morphology of the microglial cells in Tg Pl compared to Non Tg. In Tg Pl mice, we observed an enlargement of the soma size, polarization and thickening of the microglial processes, which are classical indicators of microglial activation. Minocycline treatment resulted in correction of microglial soma size (note the small, roundish morphology), with some residual thickening of processes and increase in spiny processes. **(F) **Representative micrographs illustrating the ir of Iba-1 in microglial cells in the lateral posterior thalamic nucleus of the Non Tg, Tg Pl and Tg Mino. The cells were notably smaller and less ramified than in the hippocampus, but no differences in the morphology could be observed between experimental groups. Scale bar for E and F: 20 μm. **(G**,**H) **Quantification of cell soma size and density of microglial cells from hippocampus (G) and thalamus (H) in Non Tg, Tg Pl and Tg Mino. Note that minocycline treatment resulted in significant reduction of microglial cells soma size compared to Tg Pl in the hippocampus (G). No significant differences were observed in the soma size of cells in the thalamus (H). As previously reported [23], no significant changes in microglial cell density were observed between Non Tg and Tg. This pattern was not altered across experimental groups in any area. (* *P *< 0.05, one-way ANOVA followed by Tukey post-hoc test). Non Tg: Non Tg Placebo mice; Tg Mino: Tg mice treated with minocycline; Tg Pl: Tg Placebo.

We first assessed the ability of minocycline to reduce the central nervous system (CNS) pro-inflammatory process in young, pre-plaque Tg mice by measuring levels of iNOS, COX-2 and IL-1β via western blotting (Figure 1A-C). Confirming and further expanding our previous sets of experiments [23], we found that iNOS and COX-2 were significantly up-regulated in cortical homogenates from Tg mice compared to Non Tg littermates (Figure 1A, B, *P *< 0.05). IL-1β showed a trend towards up-regulation which did not reach significance (Figure 1C). Minocycline treatment was able to inhibit neuroinflammation, as the levels of iNOS, IL-1β and COX-2 of Tg mice treated were significantly different from Tg Placebo (*P *< 0.01) but not significantly different from Non Tg controls.

Minocycline treatment did not significantly alter the levels of these markers in Non Tg animals. The following fold increases compared to Non Tg Placebo were observed in Non Tg Mino: iNOS, 1.49 ± 0.19; IL-1β, 0.99 ± 0.04; COX-2, 1.31 ± 0.15. None of these reached significance (*P *= 0.08, 0.95 and 0.31, respectively; Student's *t*-test), even though a trend was observed for the iNOS levels.

To confirm the biochemical data, we used a morphological approach to study the activation state of microglial cells. Iba-1, a structural marker for microglia [34], was used to stain brain sections from Tg and Non Tg animals. As expected, most microglial cells in Non Tg Placebo mice displayed a resting morphology, with a small soma size and symmetrical, fine arborization of the processes (Figure 1E). As previously reported, we observed an altered morphology of the microglial cells from the hippocampus of Tg Placebo animals. The cells displayed an enlarged soma size, with notable polarization and thickening of the processes, all indicative of microglial activation. In contrast, microglial cells from minocycline-treated Tg animals (Tg Mino) displayed a small, roundish soma, similar to Non Tg Placebo. Interestingly, we noticed an increase in the complexity of microglia ramification following minocycline treatment in Tg animals; the number of processes emanating from the cells appeared to be elevated and they were often thick and decorated by spines. Microglial soma size and density was measured with the assistance of the MCID 5 Image Analysis System, according to published protocols [23]. Reinforcing our biochemical results, we observed a significant down-regulation of microglial cells size in the hippocampus of Tg animals treated with minocycline (Figure 1G, *P *< 0.05) indicative of a reduced activation or reversal to a resting state. No differences in the density of microglial cells were observed, suggesting that the effect of minocycline was not mediated by a reduction in microglial proliferation.

No significant differences were found between the microglial soma size in Non Tg Placebo (38.86 μm^2 ^± 0.37) and Non Tg Mino (39.64 μm^2 ^± 1.16).

The analysis of microglial morphology was performed in the lateral posterior thalamic nucleus in the same sections (Figure 1F) as a control area which is spared by the amyloid pathology at this age [22]. In agreement with previous reports [35], we noticed that microglial cells in this area were smaller, less ramified and less dense than in the hippocampus. The analysis of microglial cells soma size and density in the thalamus revealed no differences between groups (Figure 1H). This result indicated that the effect of minocycline was specific to the hippocampus, an area burdened with intracellular Aβ-ir material.

### Minocycline treatment affects amyloid precursor protein metabolism

**Figure 2:**
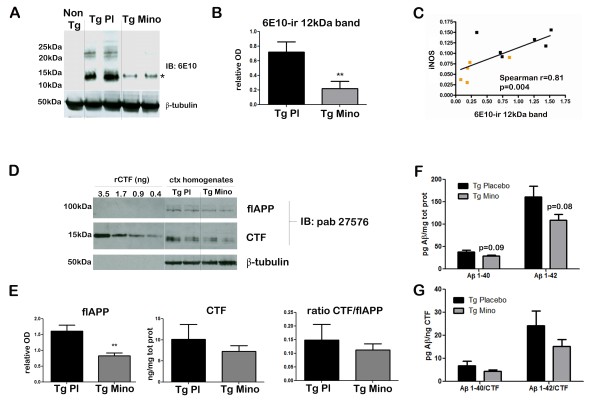
**Minocycline effects on amyloid precursor protein metabolism**. Cortical brain homogenates from Tg animals treated with vehicle (Tg Pl) or minocycline (Tg Mino) were subjected to western blotting and ELISA to determine the levels of Aβ species, full-length APP (flAPP) and CTF. **(A) **Representative western blot of cortical homogenates from Tg animals treated with vehicle or with minocycline, using 6E10 antibody. Note the strong down-regulation of the approximately 12-kDa ir band (indicated with an asterisk) which likely represents a mixture of β-CTF of APP and Aβ trimers. **(B) **Densitometric analysis of the 12-kDa band detected with 6E10. Values were normalized on neuronal specific β-tubulin. Minocycline-treated animals showed significantly lower levels compared with placebo (**P < 0.01, Student's *t*-test). **(C) **Correlation analysis of the levels of the 12-kDa band ir to 6E10 and the levels of iNOS per sample. The correlation was found to be highly significant (r = 0.81; *P *= 0.004, Spearman's correlation), where Tg Placebo animals (black dots) had the highest levels of 6E10-ir and iNOS, while Tg Mino (yellow dots) mice displayed reduced levels of ir with 6E10 and lower levels of iNOS. **(D) **Representative western blots of cortical (ctx) homogenates from placebo and minocycline-treated animals using the pab27576 antibody. This antibody recognizes an epitope on the C-terminus of APP and therefore detects two prominent bands in the blots: a high molecular weight band (about 100 kDa), corresponding to flAPP and a faster band (approximately 12 kDa) corresponded to the CTF fragments. A calibration curve using known amounts of recombinant C100 was run in the same gel for quantification purposes (the recombinant peptide ran slightly slower due to the presence of a C-terminal hexa-His tag). **(E) **Quantification of flAPP and CTF levels in cortical homogenates from Tg Placebo and Tg-treated animals. FlAPP relative optical density values were normalized on neuronal specific β-tubulin. Note the significant down-regulation of flAPP (**P < 0.01, Student's *t*-test) in Tg Mino compared with Tg Placebo. CTF absolute levels were extrapolated from the calibration curve of recombinant CTF. There was a non-significant trend towards reduction following minocycline treatment. Normalization of the relative optical density of CTF over the relative optical density of flAPP confirmed a non-significant trend towards a reduction (ratio CTF/flAPP). All data were analyzed with Student's *t*-test. **(F) **Quantification of human Aβ levels in cortical homogenates from Tg Placebo and Tg Mino using ELISA. The treatment reduced both Aβ40 and Aβ42, but the effect did not reach significance (*P *= 0.09 and 0.08, respectively, Student's *t*-test; see main text for exact values). **(G) **The ratios between Aβ (pg/mg total protein) and CTF (ng/mg total protein) were calculated per sample and compared across groups. We observed 24.29 ± 6.43 pg of Aβ42 and 15.14 ± 3.08 pg of Aβ40 per each nanogram of CTF. Minocycline treatment resulted in a reduction of the ratio (which did not reach significance), suggesting that less Aβ was produced per CTF molecule after the treatment with minocycline.

Next, we investigated the effects of the anti-inflammatory treatment on APP metabolism and Aβ levels (Figure 2). For these studies we used cortical homogenates from Tg Placebo and Tg Mino.

We first applied the commercially available monoclonal antibody 6E10, which recognizes residues 1 to 16 of human Aβ. This western blot analysis revealed several ir bands in Tg Placebo animals between 10 kDa and 50 kDa. These bands did not appear in the Non Tg homogenates, and are likely to be oligomeric forms of Aβ. Minocycline treatment resulted in the clearance of most of the 6E10-ir bands; in particular, a strong (-70%), significant down-regulation of a 12-kDa band (Figure 2A and 2B) was observed. We noticed that the levels of the 12-kDa band strongly correlated with the levels of iNOS across samples (Figure 2C, *r*= 0.81, P = 0.004, Spearman's correlation analysis). In these experimental cohorts, Tg Placebo mice displayed the highest values for both 12 kDa-6E10 and iNOS, while all but one of the Tg mice treated with minocycline displayed reduced levels of both markers. These results would suggest a link between APP metabolism and the inflammation staging.

The 12-kDa band could not be definitively identified. In fact, the 6E10 antibody is directed against the N-terminus residues of Aβ and therefore can recognize both β-CTF fragments of APP (that migrate around 12 kDa) and Aβ species (such as Aβ-trimers). To elucidate the nature of the 12-kDa band, we performed additional analysis on the homogenates that enabled us to specifically quantify the amount of CTF fragments and human Aβ.

To detect CTF fragments without cross-reactivity with Aβ, we used a specific antibody directed against the C-terminus of the APP holoprotein pab27576 (generous gift from Dr Multhaup, Freie University, Berlin [36]). This serum recognizes full-length APP (flAPP, which appeared at 100 kDa) and CTFs (which migrated around 12 kDa). In order to quantify the levels of CTF in the sample, in the same gels we included a calibration curve of recombinant CTF (Figure 2D). After this analysis, we quantified the levels of flAPP and CTF fragments (Figure 2E). We observed a significant down-regulation of flAPP relative levels (Figure 2E, *P *< 0.01) in Tg Mino animals compared to Tg Placebo. The down-regulation of flAPP was confirmed using other APP-specific antibodies such as 6E10 and 22 C11 (data not shown).

Extrapolation of the levels of CTF in the brain homogenates from the calibration curve revealed that Tg Placebo animals had 10.13 ± 3.50 ng of CTF per milligram of total protein. In the Tg Mino group, CTF fragments (both relative and absolute levels) appeared reduced (7.27 ± 1.34 ng/mg) but such reduction did not reach significance. Importantly, the decrease in CTF following minocycline treatment was not completely explained by the reduction in APP levels. In fact, when we normalized the CTF levels on flAPP levels, the trend towards a reduction was still present (Figure 2E, ratio CTF/flAPP). These results suggested that the 12-kDa band recognized by 6E10 was not exclusively constituted by CTF, as the values detected by the two antibodies were not perfectly correspondent. We therefore sought to quantify Aβ levels in the same samples.

To obtain a direct quantitative measurement of the Aβ levels we performed an ELISA analysis of human Aβ40 and Aβ42. The study revealed the presence of 160.9 ± 24.37 pg/mg total protein of Aβ42 and 38.28 ± 4.25 pg/mg total protein of Aβ40 in Tg Placebo animals. After minocycline treatment we measured 109.0 ± 13.43 pg/mg total protein of Aβ42 and 28.76 ± 2.42 pg/mg of Aβ40. Though a strong trend was observed towards reduction (*P *= 0.08 and 0.09, respectively), this did not reach significance (Figure 2F).

We then calculated the ratio between Aβ (pg/mg total protein) and CTF (ng/mg total protein) in each sample: this analysis revealed that in the Tg Placebo animals there were 24.29 ± 6.43 pg of Aβ42 and 6.76 ± 2.15 pg of Aβ40 per each nanogram of CTF. A trend towards a decrease in animals treated with minocycline was found (15.14 ± 3.08 pg of Aβ42 and 4.36 ± 0.58 pg of Aβ40 per each nanogram of CTF), suggesting fewer molecules of Aβ per molecule of CTF following minocycline treatment (Figure 2G).

### Minocycline inhibits β-site APP cleaving enzyme 1 activity in young, pre-plaque Tg mice

**Figure 3:**
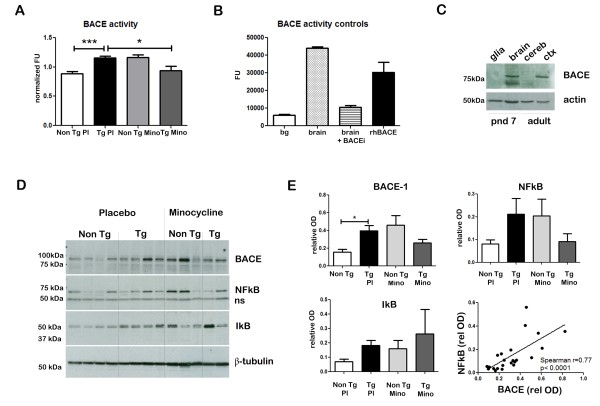
**Minocycline inhibits β-site APP cleaving enzyme 1 in young, pre-plaque Tg mice**. **(A) **BACE-1 activity was quantified from hippocampal homogenates using a well-characterized fluorometric assay. Fluorescent units (FU) data were normalized on Non Tg Placebo values. Note the significant up-regulation of BACE-1 activity in Tg Placebo compared to Non Tg Placebo (***P < 0.001), which was corrected following minocycline treatment (*P < 0.05 compared to Tg Placebo). **(B) **Specificity of the BACE-1 fluorometric assay: note how the fluorescent units detected from brain homogenates (of a Non Tg Placebo animal) are seven times higher than the background (bg). The signal was completely abolished by co-incubation with a specific BACE inhibitor (BACEi), and was in the range of activity of a recombinant human BACE protein (rhBACE, 2.5 μg). All the data were analyzed with one-way ANOVA followed by Tukey post-hoc test. **(C) **Specificity of the polyclonal BACE-1 antibody used for western blotting: the specific band (close to 75 kDa) appeared in brain extracts from post natal day 7 (pnd 7) and from adult cortex (ctx), while it was hardly detectable in cerebellum (cereb) sample and was not detectable in a glial preparation from pups. **(D) **Representative western blots of BACE-1, NFkB and IkB from the same hippocampal samples used for the enzymatic assay (ns = non-specific band recognized by the polyclonal antibody directed against NFkB). **(E) **Quantification data of BACE-1, NFkB and IkB from hippocampal homogenates. Band intensity was quantified via densitometry and the values were normalized on the neuronal specific-β-tubulin content. Note the significant up-regulation of BACE-1 levels in Tg Placebo, which was corrected by the minocycline treatment (*P *< 0.05). The levels of NFkB and BACE-1 in each sample strongly correlated (*P *< 0.001, Spearman's correlation analysis). Given the not-normal distribution of the data, the values were analyzed with Kruskal-Wallis test and Spearman's correlation analysis. BACEi: BACE inhibitor; bg; background; cereb: cerebellum; ctx: cortex; pnd: postnatal day; rhBACE: recombinant human BACE.

The strong reduction of the 12-kDa band immunoreactive with 6E10 and the altered CTF/flAPP ratio observed after minocycline treatment would indicate that the β cleavage of APP could be affected by the anti-inflammatory drug. Therefore, we proceeded to measure BACE-1 activity in the hippocampal samples, using a well-characterized fluorometric assay (Figure 3A and 3B). This kit allowed specific and robust detection of BACE-1 from brain homogenates; the fluorescent signal detected in brain homogenates was seven times higher that background, and was reduced to background levels by co-incubation with the kit BACE inhibitor (Figure 3B).

With this approach we found that Tg animals displayed significantly increased BACE-1 activity compared to Non Tg (*P *< 0.001). Supporting our biochemical findings on APP processing, we found that minocycline was able to reduce BACE-1 activity in Tg animals, and blunted it to levels that were similar to Non Tg (*P *< 0.05 Tg placebo versus Tg Mino). On the other hand, Non Tg animals receiving minocycline also displayed up-regulation of BACE-1 activity. A two-way ANOVA revealed that there was a strong interaction between the genotype and the treatment (*P *< 0.001), suggesting that minocycline exerted differential effects according to the genotype.

We used western blotting to quantify BACE-1 levels in the same samples to assess whether the effect observed was due to a pure enzymatic inhibition or to reduction in the protein content (Figure 3D). Even though this assay appeared to be less sensitive than the enzymatic activity assay, we found a significant up-regulation of BACE-1 protein levels in Tg animals compared to Non Tg (Figure 3E, *P *< 0.05). Minocycline-treated animals had intermediate levels of BACE-1, which were not significantly different from the Tg Placebo nor from the Non Tg levels. Confirming our activity data, Non Tg treated with minocycline displayed elevated levels of BACE-1 (interaction between genotype and treatment: *P *< 0.01, two-way ANOVA). The band observed was most likely specific, as it did not appear in naive glial cultures from mice pups and was barely detectable in cerebellum (Figure 3C).

Taken together, these results demonstrated that Tg animals, even prior to plaque deposition, showed increased levels and activity of BACE-1. Such up-regulation is likely to be related to an inflammatory process, as minocycline could significantly reduce BACE-1 activity and corrected BACE-1 levels.

BACE-1 and many inflammatory mediators such as iNOS, COX-2 and IL-1β are known to be under the transcriptional control of NFkB [37,38]. The active form of human NFkB is a dimer composed of the two DNA binding subunits p50 and p65. These genes are constitutively expressed in both glia and neurons and their mRNA levels are further increased in response to inflammatory signals. Dimers are normally in an inactive form, sequestered in the cytoplasm by the NFkB inhibitor, IkB (for a review, see [38]). Activation by inflammatory stimuli results in phosphorylation and degradation of IkB, translocation of NFkB into the nucleus and expression of target genes. Target genes include inflammatory cytokines, chemokines, acute phase proteins and immune receptors [37]; interestingly, IkB gene also contains NFkB binding sites [39]. We therefore proceeded to measure NFkB (p65) and IkB in the hippocampal samples (Figure 3D).

Consistent with the notion of a pro-inflammatory condition in pre-plaque McGill-Thy1-APP mice, we observed elevated levels of NFkB and IkB in the Tg Placebo animals compared with Non Tg Placebo. Treatment with minocycline in Tg animals resulted in lower levels of NFkB and higher levels of IkB. On the other hand, minocycline-treated Non Tg mice showed increased NFkB and IkB levels. None of these changes reached significance due to high variability, but we found a very strong correlation between BACE-1 levels and NFkB levels (Figure 3E, *P *< 0.001, Spearman's correlation analysis). These findings suggested that BACE-1 levels might be under the direct or indirect control of NFkB *in vivo*.

## Discussion

### Experimental design and novelty of the study

In the present studies, we aimed to elucidate the interaction between activated glia and neurons in the earliest stages of the AD pathology. Several reports in the field have documented the effect of anti-inflammatory treatments with NSAIDs in Tg models of AD-like pathology [40-42]. However, these studies were designed to assess the effect of treatment on plaque pathology, and the animals were sacrificed after plaque onset. Our approach differed from previous reports in that we wanted to clarify the role of inflammation in the early, pre-plaque stages of the pathology, which likely mimic the earliest, pre-clinical stages in AD.

This is a matter of high clinical relevance, as the available data in humans suggest a differential role of inflammation in early versus late stages of the disease. The contribution of the inflammatory process in disease onset has been highlighted by epidemiological, retrospective studies indicating a lower incidence of AD in populations receiving long-term treatment with NSAIDs [43-46]. In contrast, prospective trials applying NSAIDs to patients clinically diagnosed with AD have failed to reverse or slow down the disease, often worsening it [12-14]. Taken together, the clinical evidence is consistent with the concept that inflammation would contribute to and accelerate the AD neuropathology in its early pre-clinical stages, while it would be neutral or even beneficial in later, clinical stages. It is important to note that this hypothesis has been supported by the recent extended results from an AD anti-inflammatory preventive trial (ADAPT). The study had to be halted after only two years for safety concerns [47]; at that point in time, no beneficial effects were observed with the anti-inflammatory treatment [48]. However, the follow-up results indicated that the naproxen treatment outcome critically depended on the stage of the pathology at the moment of enrolment in the trial [49,50]. In this prospective study, in fact, an increased risk of AD onset was detected in patients who displayed some cognitive impairment (but no dementia) when they entered into the study. Such a cohort would likely represent patients closer to the disease onset. On the other hand, asymptomatic individuals treated with NSAIDs had a reduced AD incidence [49]. The concept of a differential role of inflammation at early versus late stages of the disease has been suggested for other neurodegenerative conditions, such as amyotrophic lateral sclerosis [51], and it might very well be related to the differential subsets of monocytic cells involved [8]. Therefore, the temporal window in which anti-inflammatory treatment can be beneficial and the mechanism involved in such a beneficial effect need to be further studied. We propose that pre-plaque Tg mice can be a valuable model for this type of investigation.

### Early microglial activation and its inhibition with minocycline

In agreement with our previous observations [23], we gathered biochemical (Figure 1A-C) and morphological (Figure 1D-H) evidence indicating the presence of microglial activation in the cerebral cortex and hippocampus at pre-plaque stages in the transgenic mouse model McGill-Thy1-APP.

To investigate the pathological participation of such pro-inflammatory process in the AD-like amyloid pathology, we chose to administer the tetracyclic derivative minocycline. In addition to its antimicrobial activities, this drug easily crosses the blood-brain barrier and has been shown to be beneficial in several CNS neuropathological conditions and in neurodegeneration [52]. Minocycline appears to exert its action through a plethora of mechanisms, including inhibition of key inflammatory enzymes (such as iNOS, matrix metalloprotease 9 and 5-lipoxygenase), blocking caspase-dependent and independent apoptosis and demonstrating anti-oxidant effects (for a review, see [53]).

Previous reports have studied the effect of minocycline on the full-blown amyloid pathology in APP Tg mice [28,54-56]. Overall, the drug appeared to reduce neuroinflammation and the behavioral deficits observed in Tg mice. However, in all the previous reports, the animals were sacrificed after plaque onset and the effect on pre-plaque pathology was not documented. Our study therefore represents the first report of the effect of minocycline on early, pre-plaque stages of AD-like amyloid pathology in Tg mice.

As expected, minocycline was indeed effective in reducing inflammation, as COX-2, iNOS and IL-1β levels were all found to be down-regulated in Tg animals treated with minocycline compared to placebo (Figure 1A-C). After minocycline treatment, the microglial soma size of hippocampal cells appeared to be significantly reduced (Figure 1E and 1G), along with an increase in the complexity of microglial arborization (as illustrated in the representative micrograph in Figure 1E). Both biochemical and morphological findings suggested decreased pro-inflammatory activity. Given the well-known complexity of the microglial phenotype [57], it is possible that minocycline exerted its effect by switching the microglial cells to a more neuroprotective, M2-like phenotype. Further studies will be required to pinpoint the features of microglia in response to minocycline treatment, using alternative markers such as CD45 or arginase-1 [58-60]. Interestingly, the anti-inflammatory effect of minocycline was specific to the hippocampus and the cortex, a region burdened with intracellular Aβ-oligomers [22]. In fact, microglial cells of the thalamus, an area largely devoid of Aβ material at this early age, did not appear to be affected by the treatment (Figure 1F and 1H). This result indicates that minocycline specifically interfered with a pathological inflammatory process dependent on intracellular Aβ accumulation.

### Minocycline effects on the amyloid pathology

Having assessed the ability of minocycline to inhibit the pre-plaque inflammatory process, we set ourselves to study the consequences of such anti-inflammatory treatment on the intracellular, pre-plaque phase of the amyloid pathology.

In our study, as opposed to previous reports of minocycline in AD models, the treatment was started and finished when the animals were devoid of plaques. Therefore, instead of plaque number, we focused our investigation on the cerebral levels of APP, APP-related products and soluble Aβ following minocycline treatment. Soluble levels of Aβ are particularly important indicators of the disease state, as they were shown to correlate with the degree of dementia in AD patients [61]. On the other hand, it is well established that the amyloid plaque burden does not correlate with the severity of the disease [62,63].

In this regard we first noticed that, at this early time point, inhibition of inflammation was associated with the down-regulation of APP (Figure 2). While the cellular mechanisms for such an effect remain to be determined, there is evidence in the literature that inflammatory mediators can modulate APP synthesis. IL-1β, for instance, was shown to induce APP synthesis in neurons [64,65]. The decrease in flAPP could therefore be a consequence of reduced IL-1β levels.

Besides the down-regulation of flAPP, the most significant effect we observed was the reduction of a 12-kDa band recognized with the monoclonal antibody 6E10. This band co-migrates with trimers of synthetic Aβ and is considered by some authors as oligomeric-Aβ [66,67]. However, the epitope recognized by 6E10 is shared by the C-terminus fragments of the amyloidogenic pathway (β-CTF) and 6E10 is often used to detect β-CTF from cell lysates and homogenates [68]. The fact that CTFs migrate around 12 kDa, as do Aβ trimers, complicated the interpretation of the band. To clarify the nature of this material we sought to specifically quantify CTF fragments and human Aβ in the same samples. This analysis also allowed us to determine the relative abundance of the two species in each brain.

Western blots using a specific antibody directed against the C-terminus of full-length APP (pab27576) revealed a reduction in the CTF content, which did not reach significance (Figure 2D and 2E). Even though the band recognized by pab27576 perfectly overlapped with the band seen with 6E10, the results did not fully match our analysis with 6E10. It is possible that the discrepancy is due to different specificity of the antibodies. Alternatively, some CTF material from the non-amyloidogenic pathway (αCTF which can be detected by pab27576 but not by 6E10) might have affected our quantification. We also considered the possibility that the 12-kDa band was constituted mostly of Aβ species (trimers) and so performed a highly sensitive ELISA assay for human Aβ40 and Aβ42. No plaques were detected in the animals and, from our previous study in this model, the Aβ-ir species are either monomeric or oligomeric at this stage [22]. Therefore, the Aβ material measured via ELISA can be considered as soluble in nature. Our analysis of such soluble Aβ material did reveal some degree of reduction after treatment with minocycline, but the high variability resulted in no statistical significance (Figure 2F). In summary, while the three analyses (western blot with 6E10, pab27576 and ELISA) all showed a reduction of APP-related products, the pattern observed with the 6E10 antibody was not fully reproduced by either that of CTF or Aβ alone. A likely explanation for this is that the 12-kDa band recognized by 6E10 represents a mixture of β-CTF and Aβ-oligomers. In this view, it is possible that minocycline treatment resulted in the reduction of both species, which together reached significance. Nevertheless, further investigations are needed to clarify this point.

The simultaneous presence of Aβ and APP-related products in early, pre-plaque stages of the disease in Tg models of AD is a highly controversial issue [69-73]. In particular, their relative abundance and their specific contribution to the neuropathology have not been clarified. We therefore took advantage of the data set presented here to explore the relative abundance of Aβ and CTF, and the effect of an anti-inflammatory treatment on their ratio. We compared the absolute levels of CTF (extrapolated from a recombinant CTF calibration curve, using a semi-quantitative method) and Aβ (measured via ELISA) from each sample. In Tg Placebo animals we observed (on average) 6.76 pg of Aβ40 per each ng of CTF, and 24 pg of Aβ42 per each ng of CTF. In terms of molar ratio, it appears that McGill-Thy1-APP mice harbor about 84 molecules of CTF per each molecule of Aβ40 and about 24 molecules of CTF per each molecule of Aβ42. It is therefore very likely that, while the species co-exist, β-CTF fragments represent the vast majority of the material seen with 6E10, as suggested by McAlpine *et al*. [74].

### Beta-site APP cleaving enzyme 1-deregulation in young, pre-plaque mice and its correction with minocycline

To further elucidate the effect of minocycline on APP processing, we studied the levels and activity of BACE-1, the most important β-site APP cleaving enzyme in the brain [75].

Our analysis revealed that BACE-1 levels and activity were up-regulated in the McGill-Thy1-APP Tg model, in agreement with reports from sporadic AD [76-79] and Tg models [24,80,81]. As described in young V717V Tg mice [24], BACE-1 levels and activity were up-regulated in McGill-Thy1-APP mice prior to plaque deposition. Therefore, deregulation of APP processing might be an early event in the progression of the AD-like amyloid pathology. Minocycline treatment restored BACE-1 activity to control levels, and corrected BACE-1 protein content in young, pre-plaque Tg mice (Figure 3). These results would agree with the western blotting of 6E10 (Figure 2) in indicating reduced β-cleavage of APP upon anti-inflammatory treatment.

Since the anti-inflammatory treatment with minocycline was able to correct BACE-1 up-regulation in pre-plaque Tg mice, it is very likely that the early deregulation of BACE-1 prior to plaque deposition is related to the pro-inflammatory process. This view is in line with the body of evidence indicating that neuroinflammation has a pivotal role in regulating BACE-1. In fact, several studies have indicated that BACE-1 behaves as a stress-response protein and its levels are increased by cytokines [82], oxidative stress [83], astrocytic activation [84], ischemia [85], hypoxia [86] and energy inhibition [87]. On the other hand, we did not detect any effect of minocycline treatment on the levels of Aβ-degrading enzymes such as insulin-degrading enzyme and neprilysin (data not shown).

### Minocycline mechanism of action and effect on NFkB

In an attempt to clarify the mechanism of action of minocycline, we measured the levels of NFkB, a key transcription factor which is known to regulate the expression of several inflammatory markers as well as BACE-1 and APP [38,88,89]. Increased NFkB expression is associated with neuroinflammatory conditions and it has been reported in AD [90-93]. Elevated NFkB activity was detected in Tg models of AD [94]. Interestingly, since the promoter of the IkB gene contains several NFkB binding sites [39], IkB expression is elevated in response to NFkB activation following cerebral ischemia [95] and lipopolysaccharide injections [96]. Increased levels of IkB have also been reported in AD [93].

Consistent with the results from AD samples, we found up-regulated levels of NFkB and its inhibitor, IkB, in Tg animals compared to Non Tg Placebo animals. Given the relatively high variability of the data, these changes did not reach statistical significance; however, they support the notion of a pro-inflammatory state in these brains. Accordingly, NFkB levels were reduced following minocycline treatment, while IkB was further up-regulated. A reduction of NFkB with concomitant up-regulation of IkB has been reported for other anti-inflammatory agents such as ibuprofen [97] and for glucocorticoids [98]. The increase in IkB levels is thought to further potentiate the anti-inflammatory effect, as any NFkB molecule synthesized by the cell will associate with the inhibitor and be prevented from entering the nucleus. These results, even though they did not reach significance, suggested that minocycline treatment in Tg animals resulted in an overall decreased activity of NFkB.

Furthermore, the levels of NFkB strongly correlated with BACE-1 levels in each sample. These results are consistent with the concept that BACE-1 levels and activity are tightly linked to NFkB levels *in vivo*. Even though this type of correlative analysis cannot prove causality, several indications exist that NFkB regulates BACE *in vivo*. Paris *et al*. have recently shown that the NFkB inhibitor celastrol is capable of inhibiting BACE and reducing amyloidogenic pathway in a mouse Tg model of AD [99]. Similarly, a reduction in BACE-1 and Aβ levels was found in Tg mice acutely treated with the NSAID ibuprofen [100]. This drug is endowed with multiple COX- independent mechanisms of action, including inhibition of NFkB signaling [101], peroxisome proliferator activated receptor-gamma activation [82] and gamma-secretase modulation [41]. It is very likely that, like ibuprofen, minocycline exerts its beneficial effects via multiple mechanisms of action.

Based on our results, one could speculate that the inflammation-induced hyperactivity of NFkB is responsible for the increased transcription of BACE-1 in Tg animals. This might represent a possible mechanism for the glia-to-neuron or neuron-to-glia communication in early AD, whereby the activation state of microglia can instruct the processing of APP in neurons. Alternatively, the reduction of inflammatory markers and the reduction in BACE-1 (levels and activity) following minocycline treatment might be parallel, unrelated events sharing the same up-stream events (that is, inhibition of NFkB in glia and neurons).

### Minocycline adverse effects

It is important to note that the intraperitoneal application of 50 mg/Kg/day of minocycline resulted in some toxicity: one out of eight mice (12.5%) in the Non Tg group and two out of seven mice (28%) in the Tg group died, while the remaining mice showed signs of liver toxicity and peritoneal irritation. These adverse effects precluded the completion of behavioral testing for learning and memory, such as the Morris water maze task. Liver toxicity [102] and peritoneal inflammation [103] are known side effects of intraperitoneal administration of minocycline which are seldom referred to in numerous experimental published studies. It has been established that the peripheral inflammatory process can have an impact on the microglial status in the CNS [104,105]. Indeed, the occurrence of some glial activation following minocycline treatment is supported by the rise in NFkB levels and BACE-1 activity in the Non Tg Mino group. However, these alterations were not accompanied by classical pro-inflammatory activity, as COX-2, iNOS and IL-1β were not found to be significantly different from Non Tg Placebo. As the intraperitoneal application of the drug was not inert, we cannot rule out the possibility that peripheral toxicity could have had some role in the CNS effects observed in the Tg-treated mice.

While the dose and administration route of the drug need to be optimized to avoid adverse peripheral effects, our overall results indicate that the inhibition of neuroinflammation with minocycline can be beneficial in early pre-plaque stages of AD-like amyloid pathology.

## Conclusions

This report demonstrates that the early, pre-plaque inflammatory process occurring at the initial stages of AD-like amyloid pathology can be modulated pharmacologically by the application of minocycline. The down-regulation of inflammatory markers was accompanied by a reduction of APP levels and correction of BACE-1 hyperactivity. Our results indicate that inflammation has a pivotal role in the early stages of the disease, including the modulation of APP metabolism. Interfering with inflammation could be a useful therapeutic approach in early, pre-plaque stages of AD-like amyloid pathology.

## Abbreviations

AD: Alzheimer's disease; Aβ: Amyloid-β peptide; ANOVA: Analysis of variance; APP: Amyloid precursor protein; BACE: β-site APP cleaving enzyme; CNS: Central nervous system; COX-2: Cyclooxygenase 2; CTF: C-terminus fragment; DMEM: Dulbecco's modified Eagle's medium; ELISA: Enzyme-linked immunosorbent assay; FBS: Fetal bovine serum; flap: Full-length amyloid precursor protein; HRP: Horseradish peroxidase; Iba-1: Ionized calcium-binding adaptor molecule 1; iNOS: Inducible nitric oxide synthase; IL-1β: Interleukin-1 beta; IkB: inhibitor of NFkB; Ir: Immunoreactivity; MCI: Mild cognitive impairment; NFkB: Nuclear factor kappa B; NSAID: Nonsteroidal anti-inflammatory drugs; PBS: Phosphate-buffered saline; TBS: Tris-buffered saline; Tg: Transgenic.

## Competing interests

The authors declare that they have no competing interests.

## Authors' contributions

MTF designed the experiment, carried out the intraperitoneal injections, the western blotting, the Iba-1 study of microglia morphology and BACE activity assays and drafted the manuscript. SA helped in the treatment of the animals and the collection of material for immunohistochemistry; he made substantial contributions to the interpretation of results and finalizing the manuscript. VP was involved in the treatment of the animals, performed all the perfusions and collected the material for immunohistochemistry. AD was responsible for the breeding of transgenic animals and performed all the genotyping. ACC provided intellectual guidance in the experimental design, interpretation of the results and on the editing the manuscript. All authors read and approved the final version of the manuscript.
